# An Ancient Mediterranean Melting Pot: Investigating the Uniparental Genetic Structure and Population History of Sicily and Southern Italy

**DOI:** 10.1371/journal.pone.0096074

**Published:** 2014-04-30

**Authors:** Stefania Sarno, Alessio Boattini, Marilisa Carta, Gianmarco Ferri, Milena Alù, Daniele Yang Yao, Graziella Ciani, Davide Pettener, Donata Luiselli

**Affiliations:** 1 Laboratorio di Antropologia Molecolare, Dipartimento di Scienze Biologiche, Geologiche e Ambientali, Università di Bologna, Bologna, Italy; 2 Dipartimento di Medicina Diagnostica, Clinica e di Sanità Pubblica, Università degli Studi di Modena e Reggio Emilia, Modena, Italy; University of Florence, Italy

## Abstract

Due to their strategic geographic location between three different continents, Sicily and Southern Italy have long represented a major Mediterranean crossroad where different peoples and cultures came together over time. However, its multi-layered history of migration pathways and cultural exchanges, has made the reconstruction of its genetic history and population structure extremely controversial and widely debated. To address this debate, we surveyed the genetic variability of 326 accurately selected individuals from 8 different provinces of Sicily and Southern Italy, through a comprehensive evaluation of both Y-chromosome and mtDNA genomes. The main goal was to investigate the structuring of maternal and paternal genetic pools within Sicily and Southern Italy, and to examine their degrees of interaction with other Mediterranean populations. Our findings show high levels of within-population variability, coupled with the lack of significant genetic sub-structures both within Sicily, as well as between Sicily and Southern Italy. When Sicilian and Southern Italian populations were contextualized within the Euro-Mediterranean genetic space, we observed different historical dynamics for maternal and paternal inheritances. Y-chromosome results highlight a significant genetic differentiation between the North-Western and South-Eastern part of the Mediterranean, the Italian Peninsula occupying an intermediate position therein. In particular, Sicily and Southern Italy reveal a shared paternal genetic background with the Balkan Peninsula and the time estimates of main Y-chromosome lineages signal paternal genetic traces of Neolithic and post-Neolithic migration events. On the contrary, despite showing some correspondence with its paternal counterpart, mtDNA reveals a substantially homogeneous genetic landscape, which may reflect older population events or different demographic dynamics between males and females. Overall, both uniparental genetic structures and TMRCA estimates confirm the role of Sicily and Southern Italy as an ancient Mediterranean melting pot for genes and cultures.

## Introduction

Due to their central geographic location in the Mediterranean domain, Sicily and Southern Italy hosted various human groups in both prehistoric and historic times [1], acting as an important crossroad for different population movements involving Europe, North-Africa and the Levant.

The first unquestioned colonization of Sicily has been linked to the Palaeolithic, and in particular to Epigravettian human groups coming from the mainland and entering Sicily through the present-day Strait of Messina [2]–[3]. Human remains, referable to the Upper Palaeolithic, recently discovered in Southern Italy (Grotta of Paglicci, Puglia [4]) and Sicily (Grotta d'Oriente in the island of Favignana, [5]), have been attributed to the mtDNA haplogroup HV and tentatively interpreted as descendants of the early-Holocene hunter-gatherers of Sicily and Southern Italy, who occupied this area before (Gravettian) and after (Epigravettian) the Last Glacial Maximum [5]. The transition to agriculture with the Neolithic revolution, occurred in the South-Eastern heel of Italy between 6000–5700 years BCE, then moving west towards Southern Calabria and Eastern Sicily, where traces of the same material cultures (*imprinted ceramics stentinelliane*) have been dated roughly to 5800–5400 BCE [6]. However the Neolithic pottery (*imprinted ceramics prestentinelliane*) uncovered in western Sicily (Uzzo and Kronio) are coeval (6000–5750 BCE) with the earliest occurrence of Neolithic materials in the more South-Eastern portion of the Italian Peninsula, thus suggesting potentially parallel and culturally independent processes of colonization between the eastern and western parts of the island [6].

In addition to Upper-Palaeolithic and Neolithic material cultures, historical and archaeological data offer a detailed and reliable understanding of the more recent population influences on Sicily and Southern Italy. Among the well-documented historical events, at least four main migration processes could potentially have affected the current genetic variability of the area: i) the massive occupation of Greeks (giving rise to the “*Magna-Graecia*”) started in the 8^th^ century BC from the Southern Balkans; ii) the Phoenician and Carthaginian colonization of the western part of Sicily occurred since the first millennium BC from the Levant through North Africa; iii) the Roman and post-Roman (Germanic) invasions from continental Italy and Central-Western Europe between the 300 BC and 500 AD; and iv) the more recent Muslim and Norman conquests of Sicily and Southern Italy in 8^th^–9^th^ and 11^th^–12^th^ centuries AD respectively. If on one hand the Greek colonisation of the south-eastern regions vs. the Phoenician occupation of western Sicily could have caused internal east-west cultural differentiation, on the other hand the later conquests (such as Germanic, Islamic and Norman occupations) may have contributed to reshape at different levels the genetic landscape of one of the largest Mediterranean islands, albeit their relative impacts remain still questioned.

Such a deep and complex historical stratification made the reconstruction of the genetic history and population structure of the area open to debate. Previous investigations on the genetic structure of Sicily, based on both classical, autosomal and uniparental markers, have indeed shown contrasting results about the presence [7]–[8] or the absence [9] of an east-west geographically heterogeneous distribution of genetic variation within the island [8]. By contrast, a substantial homogeneity in genetic variation, emerged from recent mtDNA-based studies focused on specific regions of Southern Italy [10]–[11]. To the best of our knowledge, all previous studies that specifically addressed the reconstruction of the genetic structure and population history of Sicily and Southern Italy, have been mostly focused on only one of the two areas at a time, moreover considering the maternal (mtDNA) and the paternal (Y-chromosome) perspectives separately.

In this study we present an high-resolution analysis of the uniparental genetic variability of Sicily and Southern Italy, by using a new accurately selected set of samples and, for the first time, by jointly analysing both paternal and maternal genetic systems at the same time. More than 300 individuals from 8 different Sicilian and Southern Italian provinces have been deeply typed for 42 Y-SNPs and 17 Y-STRs, as well as for the HVS-I and HVS-II regions and 22 coding SNPs of mtDNA. These data have been used to compare and contrast Y-chromosome and mtDNA genetic patterns within Sicily and Southern Italy, and then to investigate their affinities within the overall Mediterranean genetic landscape by further comparing our data with those of reference populations selected from Central, Western and Southern Europe, as well as from North Africa and the Levant. In this way we particularly seek to address the following questions: i) Is the genetic diversity of Sicily structured along its east-west axis and how is it patterned compared to Southern Italy? ii) Are the observed genetic patterns stratified temporally or geographically in terms of more ancient or recent peopling events, and are there any differences between maternal and paternal perspectives? iii) How is the genetic variability of Sicily and Southern Italy related to the wider Euro-Mediterranean genetic space and what are the main contributes to the current genetic pool? Since Sicily and Southern Italy have long played an important key role in the history of demic and cultural transitions occurred in Southern Europe and the Mediterranean, the clarification of these points will be of great relevance for the understanding of the different population, cultural and linguistic dynamics occurred within the whole Mediterranean area.

## Materials and Methods

### Ethics Statement

All donors provided a written informed consent to this study according to the ethical standards of the institutions involved. The Ethics Committee at the Azienda Ospedaliero-Universitaria Policlinico S.Orsola-Malpighi of Bologna (Italy) approved all procedures.

### Population sample

The genetic structure of Sicily and Southern Italy (SSI) was investigated by means of a high resolution analysis of 326 Y-chromosomes and 313 mtDNAs representing eight different SSI provinces (Figure S1). Five of these (Agrigento, Catania, Ragusa-Siracusa, Matera, Lecce) were previously published in Boattini et al. (2013) [12], whereas the remaining three (Trapani, Enna, Cosenza) were typed and analysed here for the first time. Individual samples were collected according to the standard ‘grandparents criterion’ (i.e. three generations of ancestry in the sampled province). In addition, a subsample of 129 Y-chromosomes has been selected on the basis of surnames, thanks to the availability of Italian-province-specific lists of founder surnames [13]. Due to their link with Y-chromosomes, the selection of males bearing surnames which unequivocally belong to specific places can be used to select autochthonous participants in regional population genetic studies and to obtain an “older” picture of Y-chromosomal diversity [14]. That way, we were able to simulate a putative Late-Middle-Ages sample, that is the period during which surnames spread in Italy, thus allowing to verify the effects of very recent admixture events on population genetic structure.

Blood samples (3–5 cc) were processed to extract the whole genome DNA by using a Salting Out modified protocol [15].

### Y-chromosome genotyping

PCR amplification of 17 Y-STR loci (DYS19, DYS389I, DYS389II, DYS390, DYS391, DYS392, DYS393,DYS385a/b, DYS437, DYS438, DYS439, DYS448, DYS456,DYS458, DYS635, and GATAH4) was carried out by using the AmpFlSTR Yfiler PCR Amplification Kit (Applied Biosystems, Foster City, CA) following the manufacturer's recommendations [16] in a final volume of 5 µl. The PCR reaction consisted of denaturation at 95°C for 11 min, followed by 30 denaturation cycles at 94°C for 1 min, annealing at 61°C for 1 min, extension at 72°C for 1 min, and a final extension at 60°C for 80 min. Products were sized on an ABI Prism 310 Genetic Analyzer by using the GeneScan 3.7 software (Applied Biosystems, Foster City, CA). As the Yfiler kit amplifies DYS385a/b simultaneously, avoiding the determination of each of the two alleles (a or b), these two loci were excluded from all the analyses performed. The DYS389b locus was obtained by subtracting DYS389I from DYS389II [17]. Basal haplogroups were assigned by typing the 7 SNPs (R-M173, J-M172, I-M170, E-M35, K-M9, P-M45, F-M89) implemented in the MY1 Multiplex PCR by Onofri et al. (2006) [18]. Subsequently, we explored Y-chromosome genetic variability by further typing 35 Y-SNPs. 33 of them (E-M78, E-V12, E-V13, E-V22, G-P15, G-P16, G-M286, G-U8, G-U13, I-M253, I-M227, I-L22, I-P215, I-M26, I-M223, J-M410, J-L27, J-M67, J-M92, J-M12, R-M17, R-M343, R-M18, R-M269, R-L51/S167, R-L11/S127, R-S21/U106, R-S116/P312, R-SRY2627/M167, R-S28/U152, R-M126, R-M160, R-L2/S139, R-L21/S145) were typed by using six haplogroup-specific multiplexes [19] aimed at deeply investigating the Y-markers downstream of all the major European clades (namely E1b1b1*, G*, I*, J2* and R1*). The SNP genotyping was carried out by means of PCR Multiplex amplification, followed by Minisequencing reaction based on dideoxy Single Base Extension (SBE), which was performed with the SNaPshot multiplex kit (Applied Biosystem). SBE products were analysed with capillary electrophoresis on an ABI Prism 310 Genetic Analyser. Two more SNPs (E-M81, E-M123) were finally tested with RFLP analysis, by using *HpyCH4IV* [20] and *DdeI* [21] enzymes respectively.

### Mitochondrial DNA genotyping

MtDNA genetic markers were successfully typed for 313 out of the 326 total samples. Variation at the mtDNA HVS-I and HVS-II regions was investigated by sequencing a total of 750 base pairs (bp) encompassing nucleotide positions from 15975 to 155. Polymerase chain reaction (PCR) of the HVSI/II regions was carried out in a T-Gradient Thermocycler (Whatman Biometra, Gottingen, Germany) with the following amplification profile: initial denaturation 95°C for 5 min, 35 cycles of 95°C for 30 sec, 58°C for 30 sec, 72°C for 5 min and final extension at 72°C for 15 min.

PCR products were purified by ExoSap-IT1 (USB Corporation, Cleveland, OH) and sequenced on an ABI Prism 3730 Genetic Analyzer by using a Big-Dye Terminator v1.1 Cycle Sequencing Kit (Applied Biosystems, Foster City, CA) according to the manufacturer's instructions. To reduce ambiguities in sequence determination the forward and reverse primers were used to sequence both strands of HVS-I and HVS-II regions. The CHROMAS 2.33 software was used to read the obtained electropherograms. Sequences were finally aligned to both the Revised Cambridge reference sequence - rCRS [22]–[23] and the new Reconstructed Sapiens Reference Sequence – RSRS [24] by using the DNA Alignment software 1.3.1.1 (http://www.fluxusengineering.com/align.htm).

MtDNA haplogroups were determined on the basis of diagnostic sites in the D-loop region following Phylotree mtDNA phylogeny (http://www.phylotree.org/) and confirmed with the analysis of 22 SNPs in the mtDNA-coding region by means of two PCR and one SNaPshot minisequencing reactions [25]. 17 SNPs (3010L, 3915H, 3992L, 4216L, 4336L, 4529L, 4580L, 4769H, 4793H, 6776H, 7028L, 10398L, 10400H, 10873H, 12308L, 12705L, 14766L) were those implemented in the multiplexes by Quintans et al. (2004) [26], whereas five further SNPs (3936H, 4310L, 4745L, 13708L, 13759L) were added in order to reach a finer resolution level of analysis in the mtDNA genotyping.

### Statistical Analyses

Haplogroup frequencies were estimated by direct counting. Standard diversity parameters were calculated with Arlequin 3.5.1.2 [27]. The proportion of genetic variance due to differences within or between populations was hierarchically apportioned through the analysis of molecular variance (AMOVA) implemented in the Arlequin software.

In order to set the observed genetic patterns within the Mediterranean and Southern European genetic landscape, we compared our samples with additional populations extracted from the literature (Table S1). Comparison samples were selected for representing the following key areas: North-Central Italy, Iberian Peninsula, Central Europe, the Balkans, the Levant and North Africa. As for North-African groups, literature data come mainly from urban areas, which presumptively include both Arab and Berber elements. Within each of these areas, we sought for Y-chromosome and mtDNA data (preferably but not necessarily from the same populations) that showed an in-depth resolution level comparable to our data. Sub-haplogroups were concatenated when needed for comparison purposes reaching a common level of 21 paternal and 16 maternal lineages. The number of samples bearing mtDNA and Y-chromosome reduced haplogroups within each Mediterranean population was estimated by mere counting, and relative haplogroup frequencies were computed by using the R software [28].

The correlation between geographic distances and genetic distances (Reynolds distance) based on haplogroup frequencies, was evaluated by means of a Mantel test (10,000 replications). To investigate the distribution of genetic variability within the Mediterranean Basin, Principal Component Analysis (PCA) and Spatial Principal Component Analysis (sPCA) were performed on HGs frequencies, by using the R software package *adegenet* [29]–[30]. Contrary to classic PCA where eigenvalues are calculated by maximizing variance of the data, in sPCA eigenvalues are obtained by maximizing the product of variance and spatial autocorrelation (Moran's I index) [30]. To evaluate the consistency of the sPCA-detected geographical structures versus a random spatial distribution of genetic variability, the Global and Local random tests implemented in the *adegenet* package have been applied [29]-[30]. Subsequently, to further test the significance of the genetic clusters identified by sPCA, we performed a Discriminant Analysis of Principal Components (DAPC), by using the *adegenet* package [29]–[31]. The DAPC method is aimed at describing the diversity among pre-defined groups of observations, by maximizing the between-group variance and minimizing the within-group variance. Moreover, based on the retained discriminant functions, it provides group membership probabilities of each population, which can be interpreted in order to assess how clear-cut or admixed the detected clusters are [31].

Fisher exact tests were performed on haplogroup frequencies among Mediterranean population groups, in order to determine significantly over- or under-represented HGs in any of the geographic areas considered. These tests were first performed against a background of all the Mediterranean populations by using the reduced common level of HGs resolution, and then by comparing single haplogroup frequencies of Sicily and Southern Italy with those of each comparison Mediterranean group, this time exploiting the deepest HG level available for each pairwise comparison.

The age of haplogroups (TMRCA) was estimated for those lineages found to be significantly differentiated between pairs of Mediterranean population groups, as well as focusing on the most frequent haplogroups of our dataset, due to their peculiar relevance in the genetic composition of the studied area. As for Y-chromosome time estimates, the standard deviation (SD) estimator from Sengupta et al. (2006) [32] has been used and the 95% confidence intervals were calculated based on the standard error (SE). This method does not estimate the population split time, but the amount of time needed to evolve the observed STRs genetic variation within a given haplogroup. In order to minimize the biasing effect of STRs saturation through time, all Y-chromosome age estimates were calculated selecting the eight markers with the highest duration of linearity D with time [33] and corrected for the presence of outliers as in Boattini et al. (2013) [12]. As for mutation rates, we adopted locus-specific mutation rates for each of the eight considered loci as estimated by Ballantyne et al. (2010) [34]. TMRCA for the most frequent mtDNA haplogroups was estimated by means of the ρ (rho) statistic with the calculator proposed by Soares et al. (2009) for the HVS-I region [35]. Being the molecular date estimates with ρ statistic potentially affected by past demography [36], these dates should however be interpreted cautiously. In order to avoid sampling errors, time estimates were calculated only for those haplogroups with absolute frequencies of at least 10 individuals.

The maternal and paternal genetic relationships of Sicily and Southern Italy with the other Mediterranean populations, were further addressed and compared by means of admixture-like plots based on Fst (HVS-I) and Rst (STRs) genetic distances among Mediterranean groups. Population groups were first clustered by using a non-hierarchical algorithm based on Gaussian mixture models (*mclust* R package, [37]–[38]), and then the posterior membership probabilities (for each population group to belong at each identified cluster) were calculated by using DAPC method (*adegenet* R package, [29], [31]) and graphically represented with barplots.

Finally, to formally assess on a large geographic scale, the impact of the various continental and within-continental contributions to the current Sicilian and Southern Italian (SSI) genetic variation, admixture analysis was carried out by using the mY estimator implemented in the software Admix 2.0 [39]–[40]. A special attention was paid to the selection of parental populations, due to its critical rule in obtaining appropriate estimate of admixture proportions [41]–[43]. By taking the historical and archaeological records into account, we considered the Balkans, the Levant and the North-Central Italy as putative source regions for migration processes (the latter being representative of the North-Western Mediterranean cluster identified in the Results). North Africa was excluded from the model given its negligible contribution to the current SSI genetic pool (see Results). A try-hybrid model of parental populations was therefore used to estimate the admixture rates: i) average haplogroup frequencies of North-Central Italy (SVGE, TV, BO and GRSN) for both Y-chromosome and mtDNA markers were taken as representative of the North-Central Italian parental population [NCI]; ii) data of Anatolian Greeks (PHO and SMY) and Northern Greece (NGRE) were taken as proxies for the Balkan parental population [BALK], respectively for Y-chromosome and mtDNA markers; iii) data from Lebanon (respectively LBEI, LBEK, LMOU, LNOR, LSOU for Y-chromosome and LEB for mtDNA markers) were finally taken for the Levantine parental population [LEV]. Additional information about the selected comparison populations are provided in Table S1. Finally, in order to promote reliable analysis and minimize sampling components of variance, subsets of 50 individuals were randomly selected for each putative parental group.

## Results

### Y-Chromosome perspective

The 326 unrelated individuals from 8 different locations of SSI have been assigned to 33 different haplogroups whose frequencies, for both the whole dataset as well as for each of the 8 sampling points, are detailed in Table S2. Y-STR haplotypes for the 119 newly-typed individuals are provided in Table S3. Haplogroups G-P15 (12.3%), E-V13 and J-M410* (both 9.5%), together with R-M269* (7.4%) represent the most frequent lineages found in Sicily and Southern Italy (SSI). These are followed by five R1-sublineages (R-M17, R-L2, R-P312, R-U152, R-U106), whose frequencies range from 5.2% to 3.7%, and by J-M267 which embraces almost the 5% of total variability. All these paternal lineages reportedly originated in Europe or in the Near East, whereas much lower it seems to be the African paternal contribution, mainly represented by haplogroups belonging to HG-E sub-lineages (E-V12, 2.76%; E-V22, 2.15%; E-M81, 1.53%). Contrary to what previously reported in literature [8], no differential distribution of Y-chromosome lineages has been found in our dataset. Fisher exact tests performed on HG frequencies between Southern Italy and Sicily (P-value: 0.4765), as well as between Eastern and West Sicily (P-value: 0.2998), indeed do not reveal any significant differentiation. No significant percentage of variance among groups of populations (F_CT_) has been detected by regional AMOVAs (Table S4). In the same way, when our Sicilian populations were grouped with those of Di Gaetano et al. 2009 following their East-West subdivision scheme and by using the same HG resolution level, both AMOVA (variation among groups 0.30%, P-value 0.091) and Fst index (P-value 0.094), failed to reveal any significant difference in Y-chromosome HGs composition, thus pointing out a substantial homogeneous pattern of genetic variation within the island.

Moreover, when the distribution of Y-chromosome lineages in the present-day Sicilian and Southern-Italian population has been compared with the one of the surname-based selected subset, no significant differentiation appeared (P-value: 0.9551).

High levels of within-population variability have been observed for all the 8 populations analysed, as well as for the whole dataset (Table S5), thus suggesting a high genetic heterogeneity at a micro-geographical level among the considered Sicilian and Southern-Italian populations, as confirmed also by the presence of 312 out of 326 unique STRs haplotypes. In addition, all shared haplotypes involve at most two individuals.

**Figure 1:**
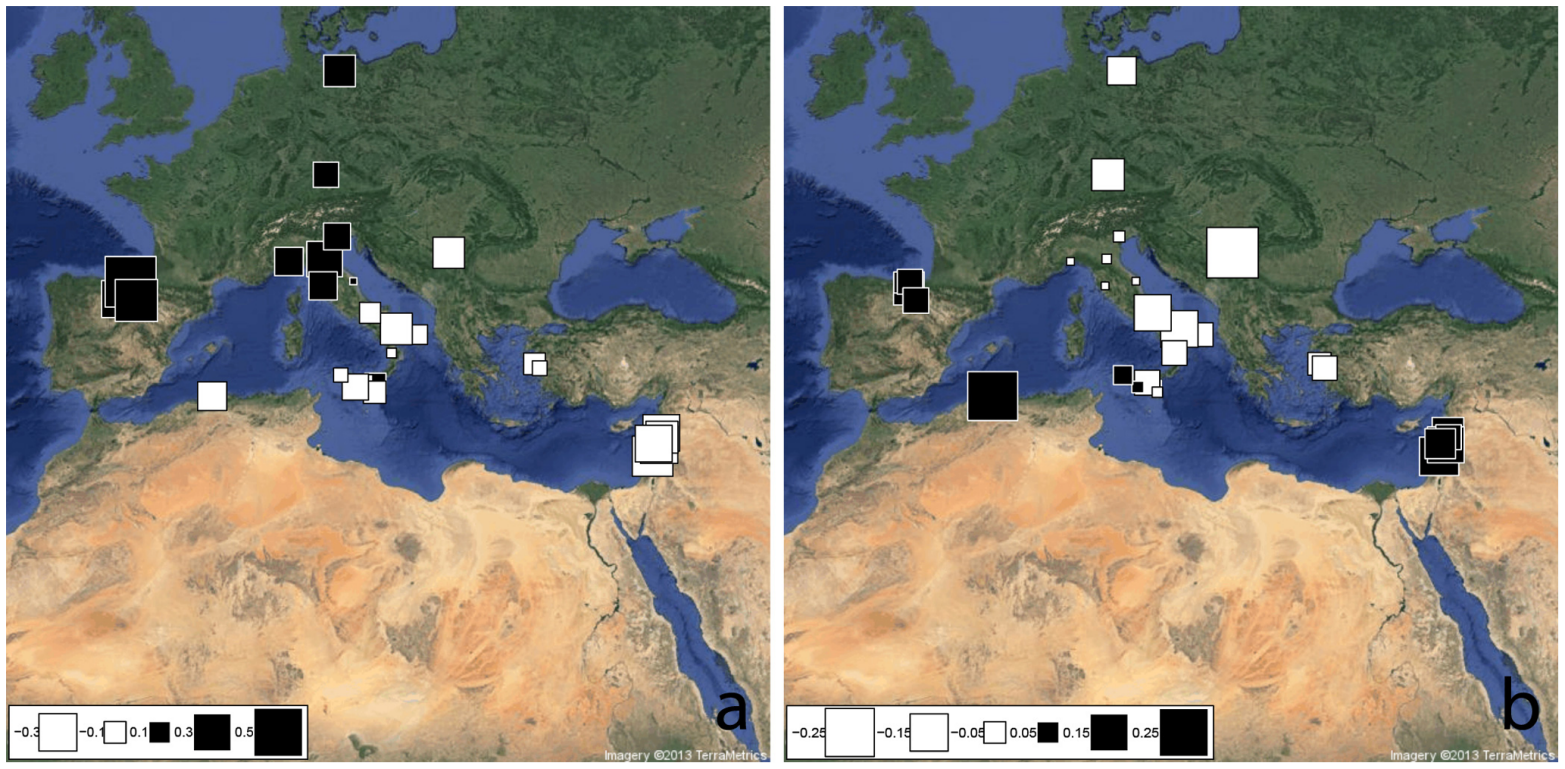
Spatial Principal Component Analysis (sPCA) based on Y-chromosome haplogroups frequencies. The first two global components, sPC1 (a) and sPC2 (b), are depicted. Positive values are represented by black squares; negative values are represented by white squares; the size of the square is proportional to the absolute value of sPC scores.

In order to more deeply explore the genetic relationships among Mediterranean groups, our samples were then compared with the 29 Euro-Mediterranean, Levantine and North-African populations extracted from the literature (Table S1), by using a common level of Y-HGs resolution. A significant positive correlation between geographical and paternal genetic distances has been observed (Mantel Test: observed value = 0.591, P-value<0.001), but no clear-cut discontinuous genetic structure was found when plotting geographical distances against the genetic ones (data not shown). However, when this general pattern of Y-chromosome HG distribution has been more deeply investigated by means of a spatial Analysis of Principal Components (sPCA), a highly significant global structure appeared (Gtest: obs = 0.146, P-value<0.001), clearly differentiating the North-Western from the Central and South-Eastern Euro-Mediterranean genetic pools (Figure 1). More precisely, the first sPC (Figure 1a) separates the Iberian, Central-European and North-Western Italian populations on one hand (black squares), from the Balkans and the Levant on the other hand (white squares). Sicily and Southern Italy particularly revealed to be well set in the genetic context of the Central and South-Eastern Mediterranean group, the only exception being Catania (CT), which instead shows a stronger affinity to the North-Western cluster (Iberian Peninsula, Germany and Northern Italy). A significant positive correlation was found between sPC1 scores and the corresponding longitudinal coordinates (R2 = 0.663, P-value<0.001), the correlation with latitudes instead being R2 = 0.440, P-value<0.001.These facts confirm the observed North-West vs. Central/South-East pattern of HGs distribution within the Mediterranean domain.

Interestingly, the second sPC (Figure 1b), despite being much less representative compared to the first one in terms of both variance and spatial autocorrelation, identifies a subdivision between the two Mediterranean coastlines, which seems to involve the Eastern and Western parts of Sicily. The first group (black squares) is indeed represented by populations from the South-Eastern Mediterranean shore (Levant and North-Africa), including also the most western Sicilian provinces (Trapani and Agrigento) and the Iberian populations. Conversely, the second cluster (white squares) is mainly a North-Eastern Mediterranean centred group, encompassing the Balkans, South-Italy and East-Sicily, together with the other central European populations. When the reliability of the sPCA-identified structures was tested by means of an AMOVA based on haplogroup frequencies, the proportion of genetic variation between groups (F_CT_) results however two times higher when grouping according to the sPC1 (8.31%, P-value<0.001) than sPC2 (4.31%, P-value = 0.004). The sPCA-suggested pattern of genetic relationships among the different Mediterranean populations, has been confirmed in the classical PCA plots reported in Figure S2a

**Figure 2:**
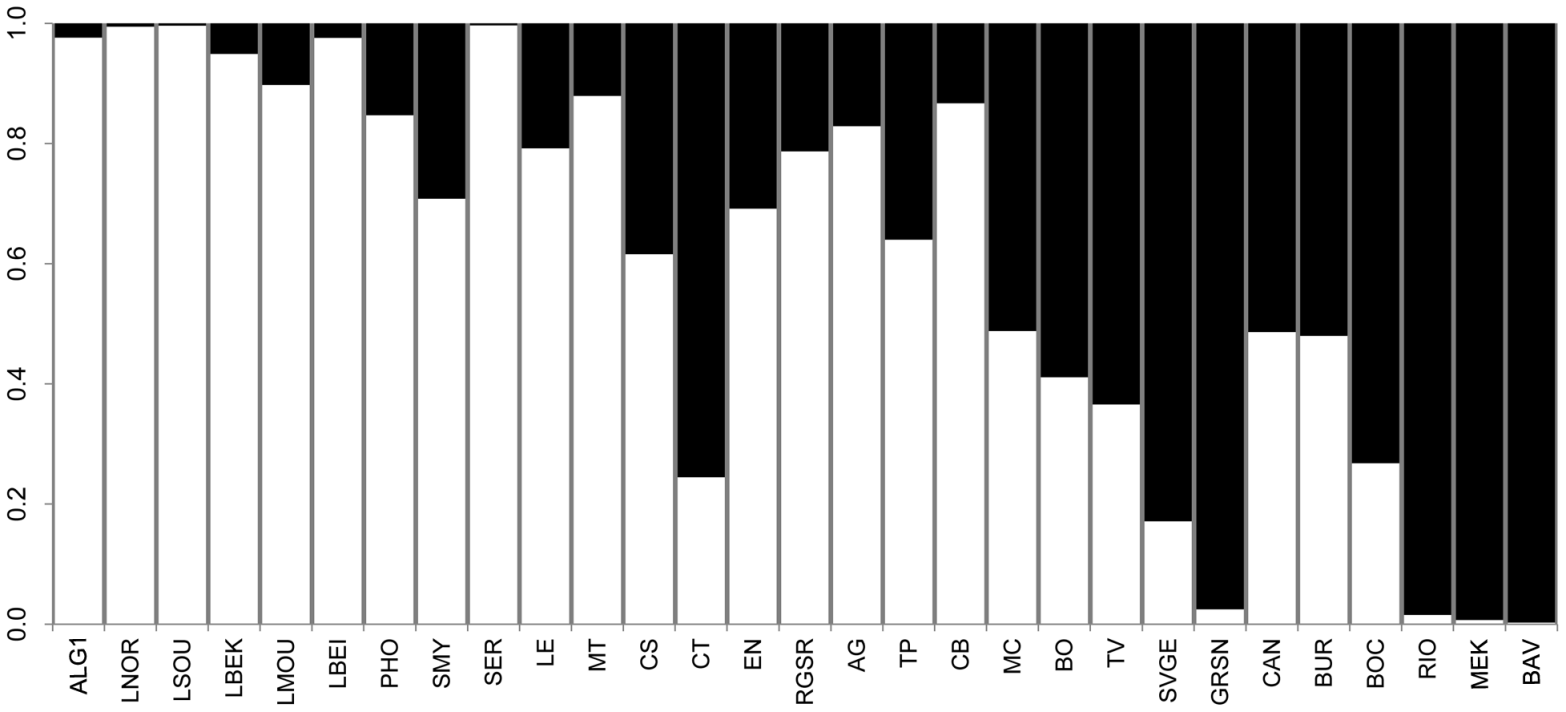
Discriminant Analysis of Principal Components (DAPC) based on Y-chromosome sPC1-identified structure. The barplot represents DAPC-based posterior membership probabilities for each of the considered populations to belong at each of the two sPC1-identified groups (white = South-Eastern Mediterranean; black = North-Western Mediterranean). Population codes as in Table S1.

The two high-structured Mediterranean clusters identified with sPC1, were further tested by means of DAPC analysis. Membership probabilities, represented with a structure-like plot (Figure 2), highlight the intermediate position of the Italian samples between the two Mediterranean clusters. In this context, Sicily and Southern Italy show clearly their stronger affinity with the populations from the South-Eastern Mediterranean side (with the partial exception of Catania - CT).

Fisher exact tests were carried out among groups of populations in order to identify significantly over- or under-represented HGs in any of the geographic areas analysed, against a background of all the other Mediterranean populations (Table S6). Haplogroup G-M201 appears significantly over-represented in the SSI genetic pool. Haplogroup R-M269, has been found significantly over-represented in Western-Mediterranean populations (IBE, GER and NCI), and under-represented in the South-Eastern Mediterranean ones (BALK, LEV and NAFR). By contrast, haplogroup J-M304(xM172) is significantly over-represented in the non-European Mediterranean shore (LEV and NAFR), being instead under-represented in European Mediterranean populations. In order to investigate further, we then performed a set of Bonferroni-corrected Chi-square tests by comparing frequencies of single lineages in SSI with those of each reference Mediterranean population group, this time exploiting the highest Y-SNP level of resolution available for each pairwise populations comparison (and considering only those lineages with absolute frequency of at least 10 individuals in SSI). Being aware that migration processes cannot be linked only with single specific haplogroups, it is however known that signals of migration should be more easily detected in more highly differentiated lineages [44]. Different haplogroups have shown significantly higher frequency in specific comparison groups than in SSI: R1b-sublineages in the western European samples (R-U152 for North-Central Italy, P-value<0.001; R-P312 for Iberian Peninsula, P-value<0.001; and R-U106 for German region, P-value<0.001), R-M17 in the Balkan Peninsula and Germany (both P-values<0.05), and J1-M267 in both Levant and North-Africa (both P-values<0.001).

As for TMRCA estimates, STR variation within the most frequent haplogroups of SSI suggests that most of them (with the exception of haplogroup G2a-P15: 9339±3302 YBP) date back to relatively recent times (Table 1), in some cases falling into time periods compatible with specific documented historical events occurred in SSI. Despite the fact that these time estimates must be taken with caution, as they might be affected by the choice of both STRs markers and their mutation rates, overall our results agree in suggesting that most of the Y-chromosomal diversity in modern day Southern Italians originated during late Neolithic and Post-Neolithic times (∼2,300 YBP for E-V13; from ∼3,200 to ∼3,700 YBP for J sub-lineages; ∼4,300 YBP for R-M17 and R-P312; and ∼2,000 YBP for R-U106 and R-U152).

**Table 1:** Age estimates (in YBP) of STR and HVS variation for the most frequent haplogroups in Sicily and Southern Italy.

Y-chromosome HG	N	%	SD	SE	TMRCA	SE
G-P15	40	12.3	373.6	132.1	9339	3302
E-V13	31	9.5	94.2	33.3	2354	832
J-M410(xM67,M92)	31	9.5	150.7	53.3	3767	1332
R-M17	17	5.2	172.2	60.9	4305	1522
J-M267	16	4.9	130.4	53.8	3261	1345
R-P312	15	4.6	175.2	61.9	4380	1549
R-U152	14	4.3	80.1	28.3	2002	708
R-U106	12	3.7	82.6	29.2	2066	730
J-M92	11	3.4	146.3	55.3	3658	1382
J-M12	11	3.4	148.6	52.6	3716	1314
J-M67	10	3.1	130.8	46.3	3271	1157

MtDNA HG	N	%	Rho	SE	TMRCA	SE
H	43	13.7	0.93	0.17	15513	5586
H1	34	10.9	0.94	0.18	15696	5768
T2	28	8.9	1.71	0.30	28589	9905
J1	16	5.1	1.50	0.38	25016	12258
HV	15	4.8	1.93	0.39	32242	12595
J2	15	4.8	1.87	0.38	31130	12434
T1	11	3.5	1.73	0.39	28806	12626
U5	11	3.5	1.64	0.39	27290	12734
H5	10	3.2	1.00	0.30	16677	9806

Standard deviation (SD) estimator (Sengupta et al. 2006) and ρ statistic calculator (Soares et al. 2009) were used for Y-chromosome and mtDNA haplogroups respectively.

### Mitochondrial DNA perspective

The maternal genetic ancestry of SSI population was explored by successfully typing both coding region SNPs and HVSI-HVSII sequences in 313 out of the 326 samples. Overall, the polymorphic sites observed in the D-loop and coding region allowed assignment of subjects to 40 mtDNA HGs (including sub-lineages), whose frequencies for both the whole dataset as well as for each of the 8 sampling points are reported in Table S2. In order to ensure the easiest access to the data [45], mtDNA sequences were deposited in the GenBank nucleotide database, under accession numbers KJ522492-KJ522611.

The observed mtDNA HGs distribution reflects the typical maternal variability pattern documented for Mediterranean Europe. In fact, most of the individuals belong to super-haplogroup H, that on the whole accounts for the 38% of the total mtDNA lineages detected in our dataset. Within H, H1 represents the most frequent sub-lineage (10.9%), followed by H5 (3.2%) and H3 (2.6%). Noteworthy is also haplogroup HV, that has been found at relatively high frequencies (4.8%). Most of the remaining samples belong to haplogroups U5, K1, J1, J2, T1, T2, thus confirming prevalent European and Middle-Eastern genetic ancestries. MtDNA haplotypes of African origin are instead represented by few haplogroups at low frequencies, namely M1 (1.3%), U6a (0.6%) and L3 (0.6%).

Within-population diversity indices reveal that, in the context of our dataset, Sicily (and particularly Western Sicily) shows slightly lower diversity values than Southern Italy (Table S5). Nevertheless, the diversity parameters observed for all the 8 populations analysed as well as for the whole dataset, fall within the range of values commonly reported in literature for both Italian and Southern European populations [11]. Similarly to Y-chromosome, mtDNA does not reveal any kind of population sub-structure both within Sicily (East vs. West Sicily) as well as between Sicily and Southern Italy, neither considering haplogroups nor haplotypes (sequences). AMOVA results show low and non-significant F_CT_ values when population samples were grouped according to geography (Table S4). Analogously, Fisher exact tests reveal no significantly different HG composition in any of the geographic regions considered (South Italy vs, Sicily, P-value: 0.5019; East Sicily vs. West Sicily, P-value: 0.0698). In the same way, both AMOVA (variation among groups 0.52%, P-value 0.082) and Fst (P-value 0.076) based on HG frequencies show the absence of significant genetic differentiation along the east-west axis of Sicily.

**Figure 3:**
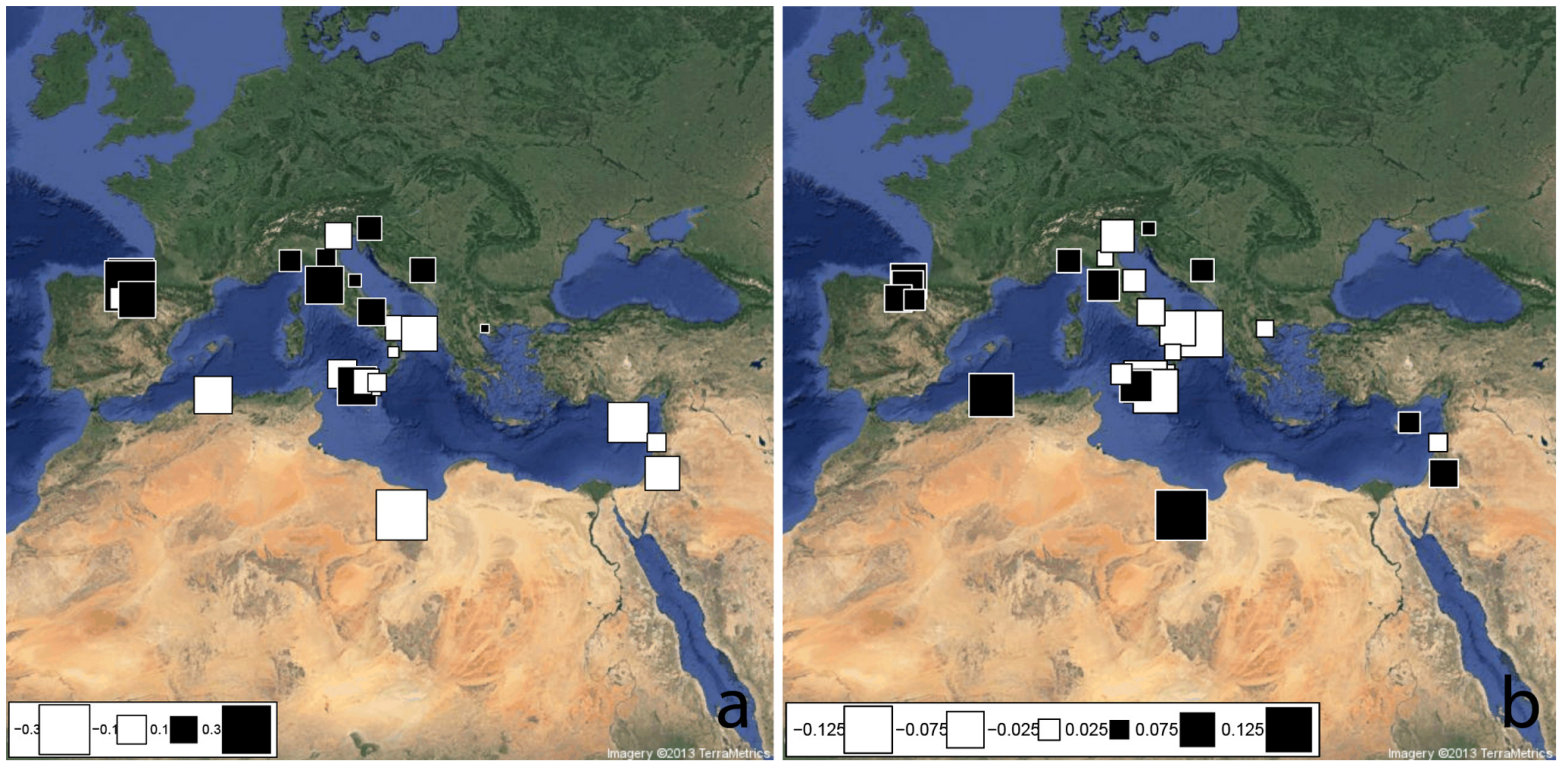
Spatial Principal Component Analysis (sPCA) based on mtDNA haplogroups frequencies. The first two global components sPC1 (a) and sPC2 (b) are depicted. Positive values are represented by black squares; negative values are represented by white squares; the size of the square is proportional to the absolute value of sPC scores.

The mtDNA HGs geographic distribution within the Mediterranean domain was investigated by comparing our sample with 26 Euro-Mediterranean, Levantine and North-African populations selected from the literature (Table S1). A Mantel test shows a low correlation between geographic and genetic distances (observed value = 0.279, P-value = 0.016). In order to further explore the relationships between geography and mtDNA genetic variability, we performed a sPCA (using HG frequencies). The highest eigenvalue obtained is the most positive one (sPC1) associated with the presence of a global structure. As previously emerged for Y-chromosome, sPC1 plot reveals a North-West/South-East (NW-SE) distribution of mtDNA genetic variation (Figure 3a). Nearly all of the Mediterranean populations (with some exceptions, i.e. AG, TV, BUR) appear indeed distributed along a longitudinal transect running from North African and Near Eastern countries (large white squares) to the Iberian Peninsula (large black squares), with the bulk of the South-Eastern European populations (including Balkans and Italy) roughly occupying an intermediate position therein (see also Figure S2b). Among them, Sicily and Southern-Italy appear linked to the South-Eastern Mediterranean coast. When the reliability of this sPC1-identified structure has been tested by means of AMOVA, the proportion of genetic variation between groups (F_CT_) results lower than in the case of Y-chromosome (2.45%) but still significant (P-value<0.001).

The second sPC (Figure 3b) highlights the position of Italy within the Mediterranean context and particularly of its South-Eastern part (large white squares). However, when tested with AMOVA, the proportion of variation between groups (F_CT_) explained by sPC2 revealed to be not significant (0.48%, P-value = 0.212). On the whole, the lack of statistical support for the global structure observed in the mtDNA sPCA (Gtest: obs = 0.165, P-value = 0.065), suggests a higher homogeneity in Mediterranean genetic variability for maternal than paternal genetic pools. Nevertheless, both uniparental markers show a similar NW-SE distribution pattern of genetic variation.

Fisher exact tests were applied to determine if differences in HG frequencies among population groups were statistically significant (Table S6). As expected, haplogroup H is found to be over-represented in Euro-Mediterranean populations and under-represented in North-African ones, while the opposite has been observed for haplogroup L. Haplogroup K is over-represented in Levantine populations, and haplogroup M in North-Africa. However, when the deepest level of HG resolution has been exploited for single pairwise comparisons between SSI and Mediterranean reference populations, we do not found any HG whose frequency is significantly higher than in our dataset. The only exception is a slightly significant (P-value: 0.045) over-representation of H1 haplotypes in the Iberian Peninsula.

Differently from Y-chromosome results, TMRCA estimates for the most frequent mtDNA haplogroups of Sicily and Southern Italy (Table 1) date back to pre-Neolithic times and could be mainly classified in lineages pre-dating the Last Glacial Maximum - LGM (∼32,200 YBP for HV; ∼31,100 YBP for J2; ∼28,900 and ∼28,600 YBP for T1 and T2; ∼27,300 for U5; and ∼25,000 YBP for J1) or dating immediately after it (∼16,700 YBP for H5 and ∼15,700 YBP for H1).

### Comparative analysis of maternal and paternal genetic pools

**Figure 4:**
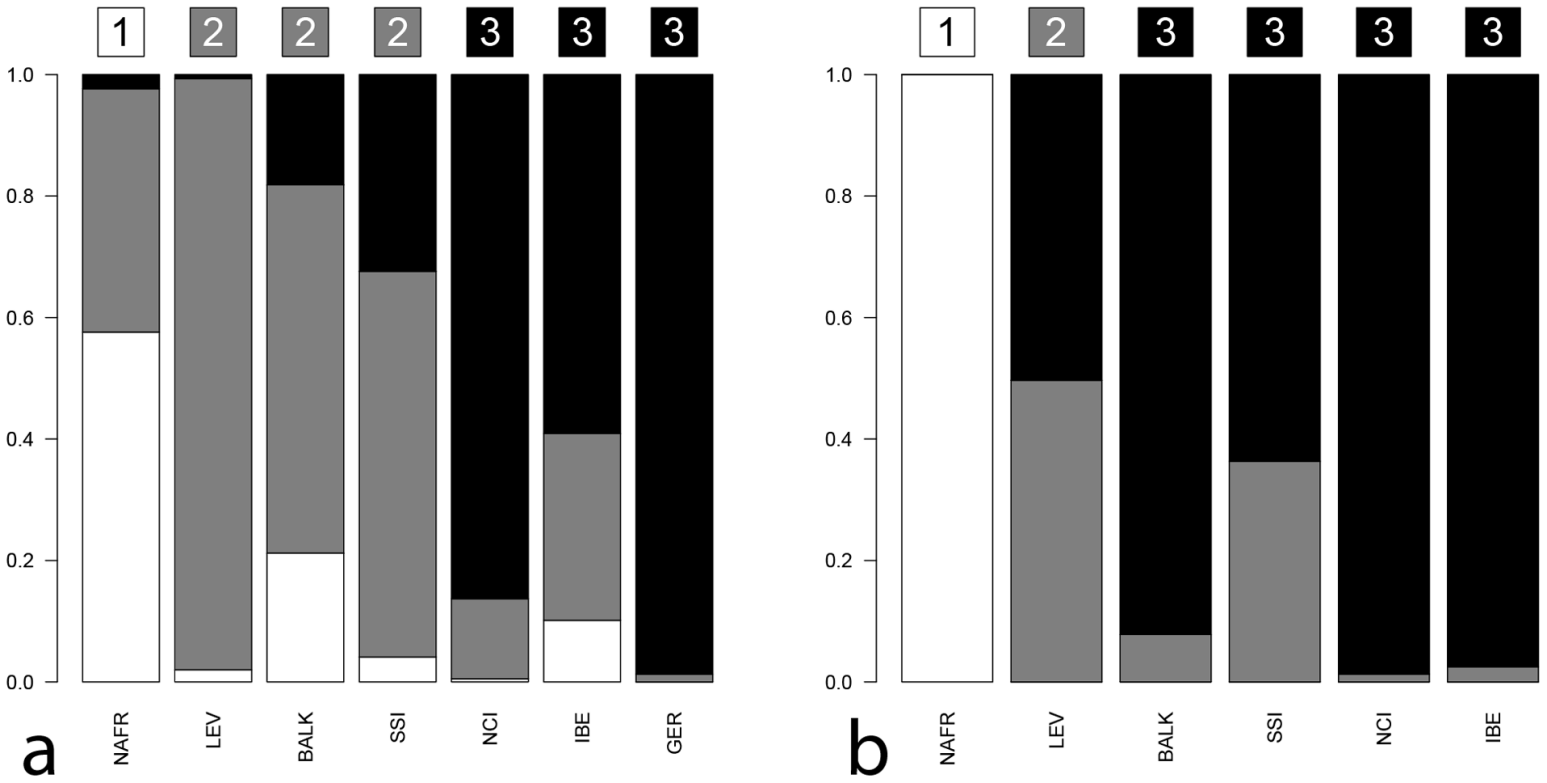
Admixture-like barplots for Y-chromosome (a) and mtDNA (b). The barplots represent DAPC-based posterior membership probabilities for each of the considered populations and for each inferred cluster (*mclust* algorithm). The affiliation of each population to a given cluster and its corresponding colour code are represented by letters (within coloured squares) on the top of each bar. Labels: NAFR: North-Africa, LEV: Levant, BALK: Balkans, SSI: Sicily and South-Italy, NCI: North-Central Italy, IBE: Iberian Peninsula, GER: Germany.

The admixture-like plot represented in Figure 4 summarizes the genetic relationships between SSI and the chosen Mediterranean populations by directly comparing Y-chromosome and mtDNA genetic results.

From a Y-chromosome point of view, SSI form a fairly coherent group with the Levantine and the Balkan populations (cluster 2), despite showing some minor contribution (black component) also from the North-Western Mediterranean group (cluster 3). From a mtDNA point of view, our results show the differentiation between European and non-European Mediterranean populations, with North Africa and the Levant clustering in separate and different groups (1 and 2). However – and differently from the other European populations – SSI shows a noteworthy contribution (grey component) from the Levantine cluster. Both genetic systems reveal a negligible contribution from North Africa (white component).

The extent of different contributions to the current SSI genetic variation was further assessed by means of an admixture analysis performed (on HG-frequencies) with the coalescent-based mY estimator implemented in the software Admix 2.0 [39]-[40]. We used a tri-hybrid admixture model, considering as source populations North-Western Italy, the Balkans and the Levant (see Materials and Methods for more details). While keeping in mind that selection of parental populations can potentially misrepresent the real estimate of admixture proportions [41]–[43], our admixture rates (Figure S3) are however quite consistent with the above-mentioned results (despite the high standard errors values). Y-chromosome admixture proportions to the current SSI genetic pool indeed confirm an high paternal contribution from the South-Eastern Mediterranean populations, and particularly from the Balkan Peninsula (∼60%), whereas about 25% of SSI Y-chromosomes can be traced back to North-Western European group. Analogously, although the present-day SSI mtDNA genetic pool is largely shared with the other South-Eastern European populations of the Mediterranean Basin (respectively Balkan and Italian Peninsulas), a remarkable proportion of maternal ancestry (especially if compared with its paternal counterpart) derives from the Levant.

## Discussion and Conclusions

Sicily and Southern Italy have long represented a natural hub for the expansion of human genes and cultures within the Mediterranean Basin [1]. Accordingly, the genetic pool of current populations inhabiting this area can be interpreted as the result of complex interplays and superimpositions between different prehistoric and more recent demographic events, ranging from the Neolithic expansion and the proto-historic Greek and Phoenician colonisations, up to the post-Roman invasions by Byzantines, Arabs and Normans. The real demographic impacts of these settlements on the population structure remain still largely uncertain based on the study of material culture and the available historical sources, and different hypotheses about the relative contributions of these events to the current gene pool composition have been proposed from a genetic point of view [7]–[9].

As a contribution to the human history of such a key area of the Mediterranean we surveyed, by means of a comprehensive evaluation of both maternal and paternal genetic landscapes, the genetic variability of a wide number of populations settled in a broad transect encompassing Sicily and Southern Italy (Figure S1). Previous reconstructions of the genetic structure of Sicily [7]–[9] focused their attention mainly on two points in the attempt to clarify its genetic history: a) the presence or absence of internal genetic differentiation along an east-west axis, and b) the extent of the genetic relationship with other populations of the Mediterranean Basin.

### Population structure and genetic history of Sicily and Southern-Italy

In contrast with previous investigations on the distribution pattern of genetic variation in Sicily [7]–[8], our results point to a substantially homogeneous composition of maternal and paternal genetic pools both within Sicily (East vs. West) as well as between Sicily and Southern Italy (Table S4). The absence of significant differences in the distribution of HG frequencies along the east-west axis of the island, as observed not only among our Sicilian populations, but also when including the samples from Di Gaetano et al. (2009) [8], provides further support to these conclusions. The comparison of the whole SSI dataset with a subset based on founder surnames, moreover suggests that the observed homogeneity in Y-chromosome composition is not the result of recent events (e.g. increased population mobility related to the social and economic changes of the 19^th^ and 20^th^ centuries); on the contrary it has been preserved at least since the initial founding and spreading of surnames in Italy. In addition, and consistently with the complex history of migration pathways and cultural exchanges characterizing the peopling history of the area, high levels of Y-chromosome and mtDNA genetic variability at both SNP and haplotype (STRs or sequence) data, have been observed in all the SSI populations here examined (Table S5).

Altogether, the high levels of within-population variability and the lack of significant genetic sub-structures fit well with the historic role of Sicily and Southern Italy as a major migration crossroad within the Mediterranean Basin. Anyway, differential contributions from the considered Euro-Mediterranean areas were observed. For instance, if the Near East, the Balkans, and – at a lesser extent – North-Western Italy probably had a relevant role in the genetic make-up of SSI, Northern African contributions seem to be almost negligible. As for the Iberian Peninsula, at present its specific genetic contribution cannot be distinguished from that of North-Western Italy, given their observed genetic similarity. These multiple migration events have probably favoured the reduction of genetic differentiation across the region, by increasing the rates of gene flows between different ethnic groups and in some cases mixing up the different genetic strata. Interestingly, the presence of massive migratory phenomena not necessarily yields genetic homogeneity in a given region. For instance, recent studies [46]–[47] showed how ethno-linguistic minorities from Sicily and Southern Italy - such as the Albanian-speaking Arbereshe - may conserve a significant genetic diversification from the rest of the population. In general, such features are more easily observed in isolated populations, thanks to their reduced population size and their cultural distinctiveness, if compared to open populations.

The patterns of genetic variability observed in our SSI sample are in agreement with the general statement that Southern European populations tend to show higher levels of genetic diversity when compared with those located at more northern latitudes [48] by virtue of the several past demographic events that affected their genetic composition over time. Additionally to the postglacial re-expansion and the demic diffusion of agriculture from Near East, also more recent events (e.g. gene flows from North Africa [48]) have been recently advocated as other possible explanations for the increased genetic diversity in the Southern European populations. Among the several historical occupations of Sicily and Southern Italy, the Pre-Roman colonisation by Greeks and Phoenicians as well as the subsequent invasions from North Africa (including the Muslim conquest, that, at least in part, was conducted by Berber forces) have been previously suggested as putative contributors to the gene pool of current Sicilian population (at least from a male perspective [8]). At this respect, the distribution of Y-chromosome haplogroup E-M81 is widely associated in literature with recent gene flows from North-Africa [49]. Besides the low frequency (1.5%) of E-M81 lineages in general observed in our SSI dataset, the typical Maghrebin core haplotype 13-14-30-24-9-11-13 [8] has been found in only two out of the five E-M81 individuals. These results, along with the negligible contribution from North-African populations revealed by the admixture-like plot analysis, suggest only a marginal impact of trans-Mediterranean gene flows on the current SSI genetic pool. Together with the Berber E-M81, the occurrence of the Near-Eastern J1-M267 in Southern-European populations has been linked to population movements from the Near East through North-Africa, and particularly as a marker of the Islamic expansion over Southern-Europe (started approximately in the 8th century AD and lasted for more than 500 years). Fisher exact tests based on HGs frequencies have revealed the presence of haplogroup J1-M267 at significantly higher frequencies in both North-Africa and the Levant than in Sicily and Southern Italy (both P-values<0.001). However, the estimated age for Sicilian and Southern-Italian J1 haplotypes refers to the end of the Bronze Age (3261±1345 YBP), thus suggesting more ancient contributions from the East. Nevertheless, our time estimate does not necessarily coincide with the time of arrival of J1 in SSI; in fact a pre-existing differentiation could potentially backdate the time estimate here obtained.

By the collapse of the Late Bronze Age societies (approximately 3200 YBP), the Mediterranean Basin underwent different waves of invasion, particularly by the Greeks of the Aegean Sea and, to a lower extent, by Levantine (Phoenicians) groups [50]. Both of them established a set of different colonies along the Mediterranean coasts of Southern Europe and North Africa. The Phoenician colony of Carthage (present-day Tunisia), given its geographic proximity to Sicily, may have played an important role in the colonization of this region. Previous Y-chromosome genetic studies on the Phoenician colonization demonstrated that haplogroup J2 in general, and six haplotypes in particular (PCS1+ through PCS6+), may potentially have represented lineages linked with the spread of the Phoenicians (“Phoenician Colonization Signal”) into the Mediterranean [51]. At this respect, it is worth noting the presence of 4 PCS+ haplotypes (namely PCS1+, PCS2+, PCS4+, PCS5+; [51]) in 9 samples of our Sicilian and Southern Italian dataset, particularly belonging to haplogroups J1-M267 (n = 2), J2-M410* (n = 1), J2-M67 (n = 5), and J2-M12 (n = 2). However, sub-lineages of haplogroup J2 have been also associated with the Neolithic colonization of mainland Greece, Crete and Southern Italy [52], and our TMRCA estimates for J2-subhaplogroups (ranging from 3271±1157 YBP to 3767±1332 YBP) cannot exclude an earlier arrival of at least some of the J2 chromosomes in Sicily and Southern-Italy during Neolithic times.

On the other hand, Y-chromosome lineage E-V13 is thought to have originated in southern Balkans [53]–[54] and then to have spread in Sicily at high frequencies with the Greek colonization of the island [8]. The E-V13 core haplotype 13-13-30-24-10-11-13 (DYS19-DYS389I-DYS389II-DYS390-DYS391-DYS392-DYS393), which define the southern Balkan Modal Haplotype and reaches frequencies of ∼12% in continental Greece [52], has been found in 10 out of the 31 E-V13 samples of Sicily and Southern Italy. This result, along with the high frequency of E-V13 lineages generally observed in our dataset (the second most frequent haplogroup after G2a), confirms the presence of gene flows into Sicily from the Balkans as previously observed by Di Gaetano et al. (2009) [8]. Accordingly, our TMRCA estimate for E-V13 (2354±832 YBP) agrees with the results previously reported in literature for the Sicilian population (2380 YBP, [8]). Altogether, these results do not exclude the possible introduction of some of these Y-lineages with migration processes originated in the Balkans and particularly associated with the Greek colonisation of Southern Italy.

Y-chromosome haplogroup G2a-P15 turn out to be of particular interest in the paternal genetic make-up of Sicily and Southern Italy. Its older age estimate (9339±3302 YBP) – if compared to those of other haplogroups – along with its significantly over-represented frequency in SSI, are consistent with the hypothesis recently suggested by Boattini et al. (2013) [12] according to whom this lineage could be a possible candidate for a pre-Neolithic ancestry in Italy. However the CIs of our time estimate cannot exclude alternative hypotheses such as a diffusion of its major sub-clades during Neolithic and Post-Neolithic times, as recently discussed by Rootsi et al. 2012 [55].

Contrarily to Y-chromosome results, age estimates for mtDNA haplogroups suggest that most of the maternal diversity of the current Sicilian and Southern Italian population is composed by lineages present in Europe as early as the LGM (Table 1). The Late Glacial and Postglacial re-occupation of Europe from refugial areas located in the Mediterranean Peninsulas, has played a major role in shaping the gene pool of modern Europeans [56] and some of the differences in genetic diversity of current European populations have been attributed also to this process [48]. Consistently, the geographic distribution and ages of some mtDNA haplogroups, such as V, H1 and H3, have been associated to events of postglacial re-colonisation from Southern European glacial refugia, and particularly from the Franco-Cantabrian area [57]–[60]. Further evidences of post-glacial resettlement from Southern refugia have been recently suggested also for the mtDNA haplogroup H5 (the third most common European H-sublineage after H1 and H3), if considering its higher occurrence in southern European populations (particularly Italy) and its evolutionary age ranging approximately between 11,500 and 16,000 YBP [61].

Together with the Iberian and Balkan peninsulas, also Italy and particularly SSI might have played an important role during the post-glacial re-expansion, as widely attested by several animal and plant species [62]–[68]. As in the case of Iberia and the Balkans, the presence of numerous Epigravettian sites suggests that Italy could have acted as such also for humans [69], despite the fact that strong genetic evidences are still missing (except for mtDNA haplogroup U5b3 [70]).

Haplogroups H1 and H5 appeared to represent the most frequent H-sublineages in SSI, and their age estimates (Table 1) are consistent with post-glacial time periods, as previously observed for both Southern Italy [11] and the entire Peninsula [12]. Nevertheless, a significant (P-value 0.045) over-representation of H1 haplotypes and an older age (17295±5119 YBP) has been obtained for the Iberian population (as represented by the considered reference samples) than in our SSI datatset, thus suggesting, at least for H1,a post-glacial re-expansion presumptively originated in the Franco-Cantabrian area.

Interestingly, mtDNA haplogroup HV confirmed to be the most ancient lineage in Sicily and Southern Italy, predating the LGM (32242±12595 YBP) and thus representing a possible candidate for the Palaeolithic ancestry of Southern Italy, even though possible post-LGM expansions of its major sub-branches should be taken into account as potentially affecting the time estimates here obtained. Further analyses, involving the complete sequencing of mtDNA genomes and the analysis of ancient DNA samples, are therefore needed in order to more deeply address this point and to confirm the relevance of this haplogroup in the first peopling of Sicily by moderns humans, as recently suggested by some Palaeogenetic researches [5].

### Patterns of genetic relationships within the Mediterranean Basin

When comparing SSI with Mediterranean reference populations, Y-chromosome results (Figure 1 and Figure S2) revealed a clear-cut genetic differentiation between the North-Western vs. the Central- and South-Eastern Mediterranean genetic pools (as confirmed by both sPCA G-test and AMOVA F_CT_ statistically significant tests). These results are consistent with our previous study about Italy [12], in which we detected a discontinuous paternal genetic structure, clearly separating the South-Eastern and the North-Western parts of the Italian Peninsula. Here this pattern appears extended to the whole Mediterranean Basin, particularly suggesting a shared genetic background between South-Eastern Italy and the South-Eastern Mediterranean cluster from one side, and between North-Western Italy and the Western Europe from the other side (Figure 2).

Y-chromosome results however contrast with the lack of statistical support to the sPCA global structure observed for mtDNA diversity, excepted for a similar NW-SE genetic pattern identified by sPC1 (Figure 3). The common South-East to North-West pattern in the distribution of genetic variation across the European and Mediterranean domain, could be interpreted as reflecting the same SE to NW genetic cline extensively reported in literature for the whole of Europe [71]–[74]. However, the general lack of statistical support to the global structure observed for mtDNA markers suggests a higher homogeneity for maternal than paternal genetic pools in the Mediterranean genetic landscape. These results could be ascribed to older population events and/or different demographic and historical dynamics for females than males. The differential income of male genes into a population has been indeed advocated as one of the possible reasons why matrilines tend to be more stable over time than patrilines. Such a male-biased pattern has been suggested for the Neolithisation of Southern Europe [75]–[76] and proposed also in the case of the first Greek incoming groups in Sicily and Southern Italy [77]. As a consequence of such kind of sex-biased dynamics, male lineages could be better suited to detect more recent population events than the female ones, which instead trace back to more ancient time periods [49]. Accordingly, while the time estimates for Sicilian and Southern Italian mtDNA haplogroups date almost unanimously to Pre-Neolithic times, Y-chromosome results highlight the importance of Neolithic and Post-Neolithic (Metal Ages) demographic events in shaping the current paternal diversity composition (Table 1). Moreover, differences between the two uniparental genetic systems also appeared when the genetic relationships among Mediterranean population groups were more deeply addressed in admixture analyses (Figure 4 and Figure S3). In fact, whereas the different continental and within continental contributions to the current SSI genetic pool appeared to be more equally distributed on the maternal side (despite a noteworthy contribution of Levantine females), the paternal counterpart appeared to be clearly affected by South-Eastern Mediterranean, mainly Balkan, males.

In summary, Sicilian genetic diversity revealed to be not structured along the east-west axis of the island; on the contrary both maternal and paternal genetic markers suggest an homogeneous genetic composition both within Sicily, as well as between Sicily and Southern Italy. These results are consistent with the largely shared genetic histories of the Southern Italian populations, and reflect their historical and archaeological role as a major Mediterranean ‘melting pot’ where different peoples and cultures came together over time, albeit with different contributes depending from the source area.

When Sicilian and Southern Italian population were contextualized within the Mediterranean domain, the observed homogeneous pattern of genetic variation, however revealed different temporal dynamics and spatial genetic contributions to the maternal and paternal inheritances,.

Besides a common SE-NW distribution pattern of genetic variation, mtDNA indeed suggests an homogeneous genetic landscape related to older populations events and/or higher female mobility. On the contrary, Y-chromosomal genetic diversity appears significantly differentiated between a Central/South-Eastern and a North-Western Mediterranean group, the Italian Peninsula occupying an intermediate position between them. In particular, and consistently with the most recent syntheses on the Italian genetic structure based on both uniparental markers [12] and genome wide data [78], Sicily and Southern Italy exhibit predominant influences from the Central and South-Eastern Mediterranean regions, especially the Balkans. If contacts between SSI and the Balkans date back at least to the Neolithic, the Greek dominion of the late Metal Ages seems to have played a particularly important role, accounting at least in part for the observed shared genetic background between SSI and the Balkan Peninsula. Further studies involving model-like populations such as ethno-linguistic minorities, together with wide-genome analyses, will provide a complementary overview to the perspectives offered by uniparentally-inherited markers, thus allowing to more deeply test specific hypotheses related to the peopling history of Sicily and Southern Italy. In addition, this will represent the starting point for future explorations aimed at specifically investigating the impact of different historical, geographical and linguistic factors on the population genetic substratum, within specific macro- and micro-geographic contexts of the Euro-Mediterranean genetic landscape.

## Supporting Information

Figure S1 **Geographic map showing the location of the eight populations analysed in the present study.** The table at the bottom right details the set of provinces (sampling points) and the number of samples successfully typed for both Y-chromosome and mtDNA markers. (Map modified from Wikipedia, http://en.wikipedia.org/wiki/File:Southern_Italy_topographic_map-blank.png).
(TIF)

Figure S2 **Principal Component Analysis (PCA) based on haplogroup frequencies for Y-chromosome (a) and mtDNA (b).** Population codes as in Table S1. Colour codes for geographic affiliations as in the legends at the bottom-left of each plot. Legend abbreviations: NAFR: North-Africa, LEV: Levant, BALK: Balkans, SSI: Sicily and South-Italy, NCI: North-Central Italy, IBE: Iberian Peninsula, GER: Germany.
(TIF)

Figure S3 **Estimated admixture contributions (mY estimator) from three parental populations to the current population of Sicily and Southern Italy for Y-chromosome (left) and mtDNA (right).** Color codes: South-Western Europe (blue), the Balkans (yellow) and the Levant (green). Error bars represent standard deviations calculated on the basis of 10,000 bootstraps.
(TIF)

Table S1 **List of the selected Mediterranean populations used for Y-chromosome and mtDNA comparative analyses.**
(XLSX)

Table S2 **Y-chromosome and mtDNA haplogroup frequencies for the whole Sicilian and Southern Italian dataset and for each population analyzed.** For each Y-chromosome lineage the absolute number of individual and the percentage frequency (between brackets) are reported.
(XLSX)

Table S3 **Y-Chromosome STRs haplotypes and SNPs analysis results for the newly-typed samples of the present study (N = 119).**
(XLSX)

Table S4 **Analyses of the molecular variance (AMOVA) for Y-chromosome and mtDNA based on both haplogroup frequencies (SNPs) and haplotype data (STRs or sequences).**
(XLSX)

Table S5 **Diversity parameters for uniparental genomes based on haplogroup frequencies (SNPs) and haplotype data (STRs or sequences).**
(XLSX)

Table S6 **Fisher exact test for Y-chromosome and mtDNA HG frequencies among the Mediterraean population groups.**
(XLSX)
