# Supplementary figures and images for: An Ancient Mediterranean Melting Pot: Investigating the Uniparental Genetic Structure and Population History of Sicily and Southern Italy
Source: PLoS One. 2014 Apr 30;9(4):e96074. doi: 10.1371/journal.pone.0096074 (PMC4005757; doi:10.1371/journal.pone.0096074)

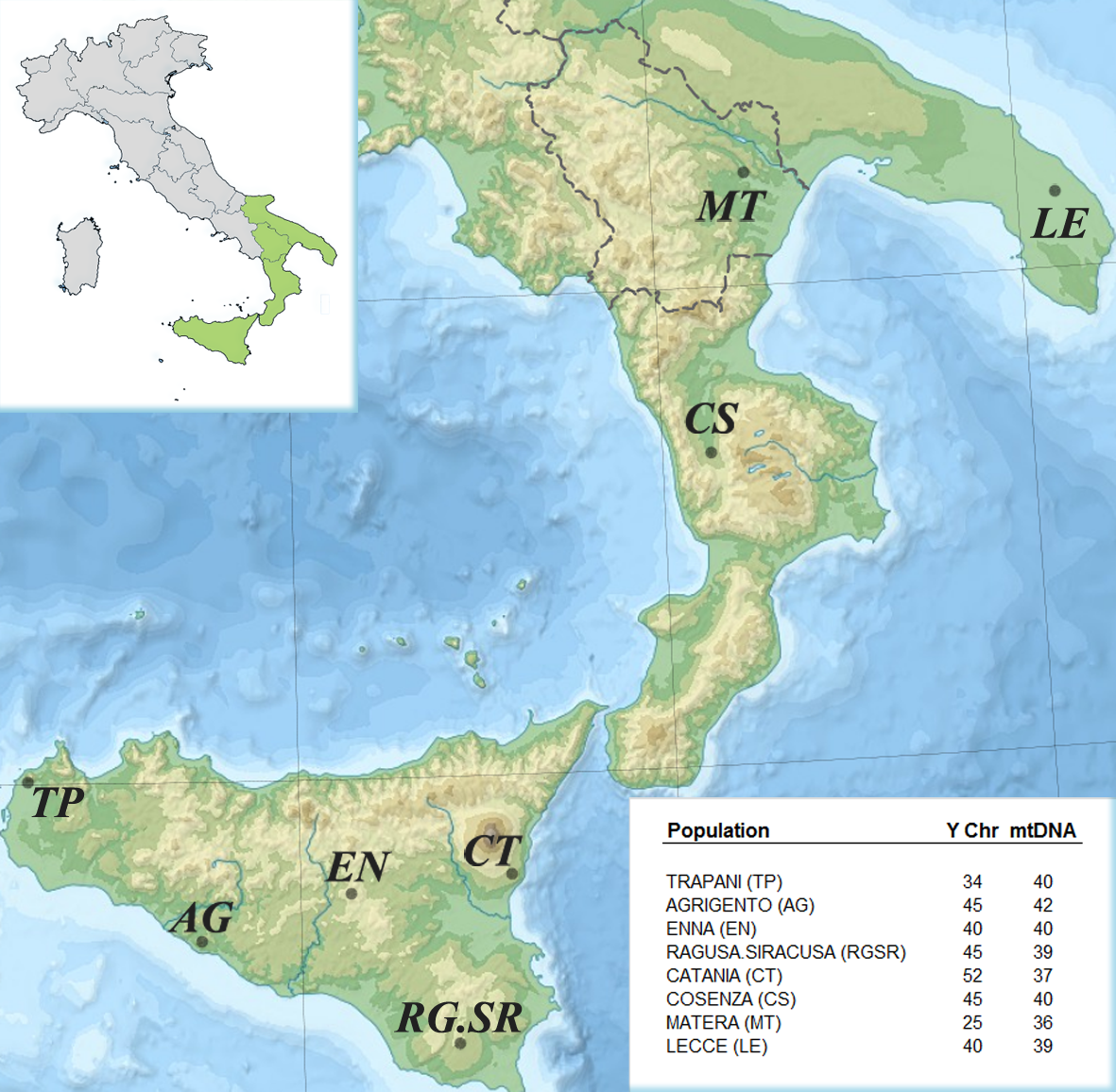

Supplement: Figure S1 — Geographic map showing the location of the eight populations analysed in the present study. The table at the bottom right details the set of provinces (sampling points) and the number of samples successfully typed for both Y-chromosome and mtDNA markers. (Map modified from Wikipedia, http://en.wikipedia.org/wiki/File:Southern_Italy_topographic_map-blank.png). (TIF) [file pone.0096074.s001.tif]

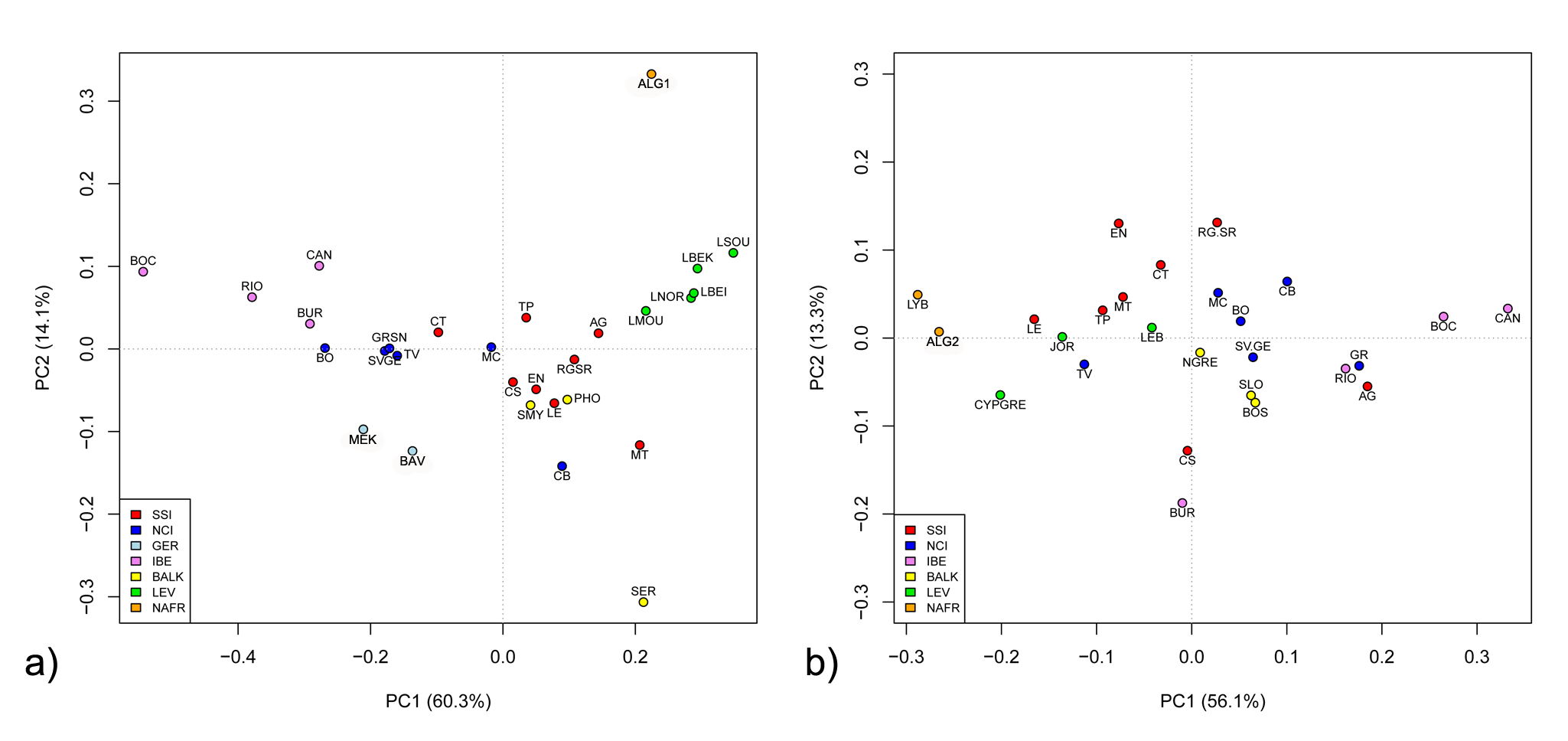

Supplement: Figure S2 — Principal Component Analysis (PCA) based on haplogroup frequencies for Y-chromosome (a) and mtDNA (b). Population codes as in Table S1. Colour codes for geographic affiliations as in the legends at the bottom-left of each plot. Legend abbreviations: NAFR: North-Africa, LEV: Levant, BALK: Balkans, SSI: Sicily and South-Italy, NCI: North-Central Italy, IBE: Iberian Peninsula, GER: Germany. (TIF) [file pone.0096074.s002.tif]

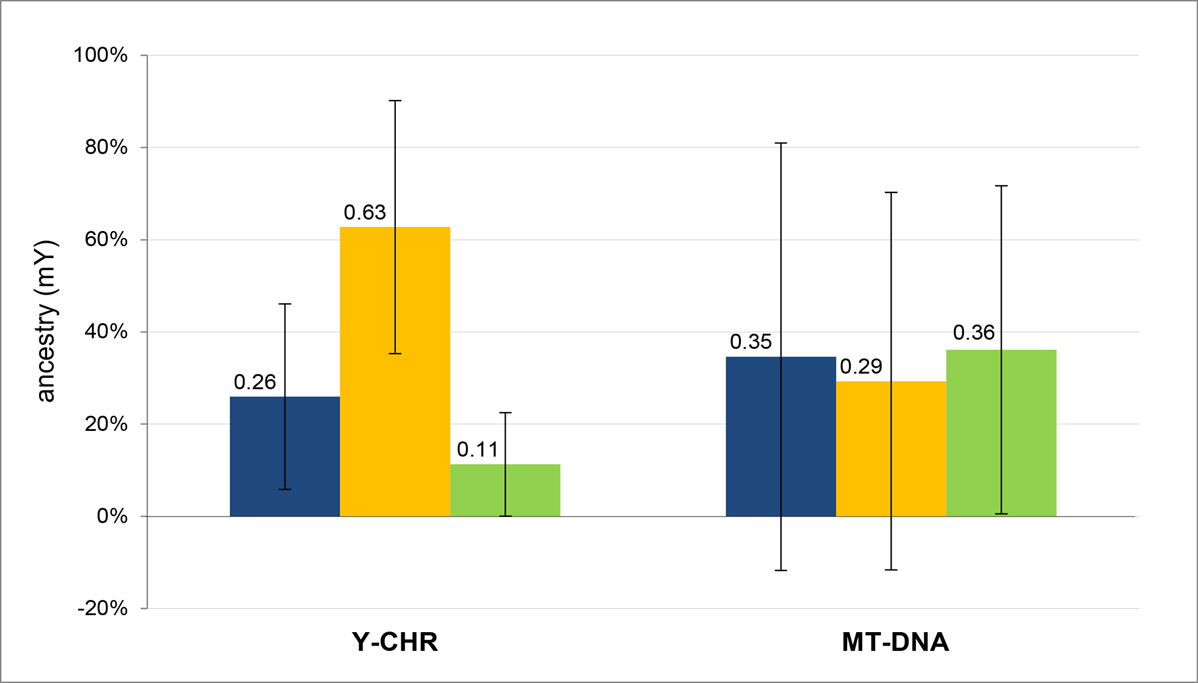

Supplement: Figure S3 — Estimated admixture contributions (mY estimator) from three parental populations to the current population of Sicily and Southern Italy for Y-chromosome (left) and mtDNA (right). Color codes: South-Western Europe (blue), the Balkans (yellow) and the Levant (green). Error bars represent standard deviations calculated on the basis of 10,000 bootstraps. (TIF) [file pone.0096074.s003.tif]
